# Engineered acetogenic bacteria as microbial cell factory for diversified biochemicals

**DOI:** 10.3389/fbioe.2024.1395540

**Published:** 2024-07-11

**Authors:** Jun-Zhe Zhang, Yu-Zhen Li, Zhi-Ning Xi, Hui-Peng Gao, Quan Zhang, Li-Cheng Liu, Fu-Li Li, Xiao-Qing Ma

**Affiliations:** ^1^ Qingdao C1 Refinery Engineering Research Center, Qingdao Institute of Bioenergy and Bioprocess Technology, Chinese Academy of Sciences, Qingdao, China; ^2^ University of Chinese Academy of Sciences, Beijing, China; ^3^ Sinopec Dalian (Fushun) Research Institute of Petroleum and Petrochemicals, Dalian, China; ^4^ Key Laboratory of Marine Chemistry Theory and Technology (Ministry of Education), College of Chemistry and Chemical Engineering, Ocean University of China, Qingdao, China; ^5^ Shandong Energy Institute, Qingdao, China; ^6^ Qingdao New Energy Shandong Laboratory, Qingdao, China

**Keywords:** acetogenic bacteria, synthetic biology, metabolic engineering, C1 gases, gas fermentation, cell factory

## Abstract

Acetogenic bacteria (acetogens) are a class of microorganisms with conserved Wood-Ljungdahl pathway that can utilize CO and CO_2_/H_2_ as carbon source for autotrophic growth and convert these substrates to acetate and ethanol. Acetogens have great potential for the sustainable production of biofuels and bulk biochemicals using C1 gases (CO and CO_2_) from industrial syngas and waste gases, which play an important role in achieving carbon neutrality. In recent years, with the development and improvement of gene editing methods, the metabolic engineering of acetogens is making rapid progress. With introduction of heterogeneous metabolic pathways, acetogens can improve the production capacity of native products or obtain the ability to synthesize non-native products. This paper reviews the recent application of metabolic engineering in acetogens. In addition, the challenges of metabolic engineering in acetogens are indicated, and strategies to address these challenges are also discussed.

## 1 Introduction

The functioning of modern society and industry heavily relies on energy, fuels, and chemicals, most of which are primarily derived from fossil fuels. The non-renewability of fossil fuels poses a potential shortage risk, and their extensive usage results in the emission of greenhouse gases, which contributes to global climate change. Renewable energy and chemicals can serve as alternatives, offering a potential solution to current environmental challenges (Jiang et al., 2021). In conventional biofuel production, food crops (first generation) or cellulosic biomass (second generation) are typically used as substrates, with microorganisms employed in the fermentation process to produce ethanol, butanol, and other chemicals (Atsumi et al., 2008a; Atsumi et al., 2008b; Lin et al., 2015; Tian et al., 2017; Birgen et al., 2019; Jeswani et al., 2020; Riaz et al., 2022). However, the production of feedstocks for first-generation biofuels relies heavily on agricultural land, which increases the risk of exacerbating food scarcities. On the other hand, second-generation biofuels using lignocellulose or waste materials as feedstocks are more sustainable and environmentally friendly. However, the feedstocks require pre-processing steps, such as degradation, making the process inefficient and costly (Xu et al., 2018). Capture and utilization of gaseous one-carbon (C1) waste feedstocks using carbon oxide (CO and/or CO_2_) fixing microorganisms through gas fermentation has emerged as a promising route, holding both industrial and environmental significance. Acetogenic bacteria (acetogens) are promising platform microorganisms for C1 gas fixation. They are a class of obligate anaerobic Gram-positive bacteria, that can utilize CO_2_/H_2_ or CO as energy and carbon source for autotrophic growth (Drake et al., 2006; Drake et al., 2008). The Wood-Ljungdahl pathway (WLP) is employed for CO_2_ fixation and acetyl-CoA production, and is coupled with energy conservation (Drake et al., 1997; Ragsdale and Pierce, 2008). Acetyl-CoA serves as a precursor for biomass synthesis or for the generation of specific products, such as acetate and ethanol. So far, over 100 acetogenic species in 23 genera have been identified that grow under a wide range of temperature and pH (Drake et al., 2006; Lee et al., 2022) Depending on the species, in addition to acetate, diverse biochemicals, including ethanol, 2,3-butanediol, lactate, or other acids and alcohols can be produced from C1 feedstocks (Kim et al., 2023). However, not all the acetogens have been investigated in details. Species from the genera of *Clostridium*, *Acetobacterium*, and *Moorella* are currently the most extensively studied for developing industrial applications.

In this review, the physiology and metabolism of acetogens focusing on WLP and energy conservation is firstly introduced. Recent progress in the development of genetic manipulation approaches and achievement of metabolic engineering in acetogens are summarized. Furthermore, the challenges of metabolic engineering in acetogens are outlined, and strategies aimed at overcoming these challenges are also discussed.

## 2 Wood-Ljungdahl pathway and energy conservation

### 2.1 Wood-Ljungdahl pathway

**FIGURE 1:**
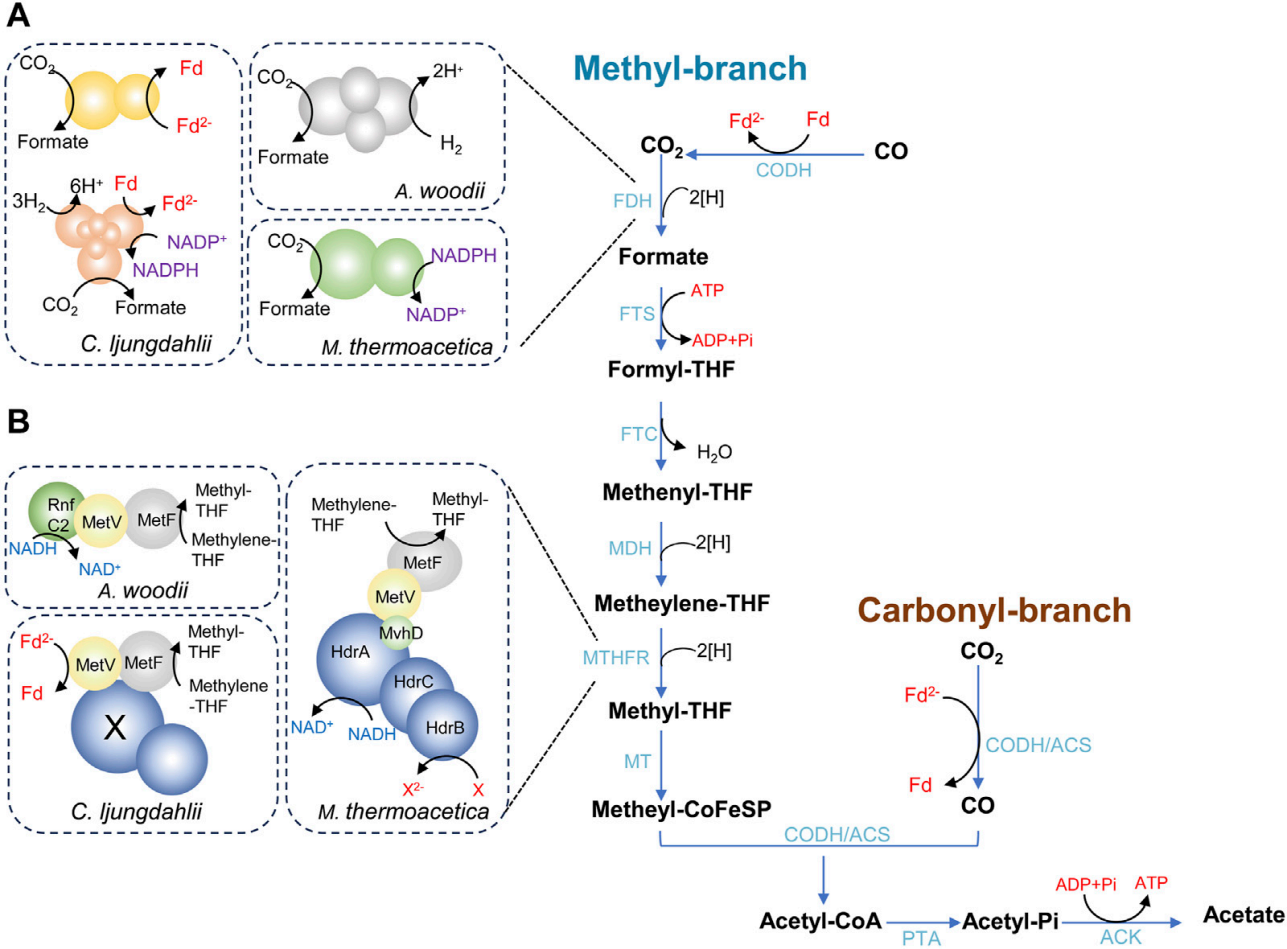
The Wood-Ljungdahl pathway (WLP) in acetogens. **(A)** shows the diversity of formate dehydrogenases; **(B)** shows the diversity of methylene-THF reductases. ACK, acetate kinase; ACS, acetyl-CoA synthase; CODH, CO dehydrogenase; FDH, formate dehydrogenase; FTS, formyl-THF synthetase; FTC, formyl-THF cyclohydrolase; MDH, methylene-THF dehydrogenase; MTHFR, methylene-THF reductase; MT, methyltransferase; PTA, phosphotransacetylase; THF, Tetrahydrofolate.

WLP is the key pathway for acetogens to fix CO_2_ and generate acetate, as illustrated in Figure 1 (Ljungdahl, 1986; Wood et al., 1986). WLP consists of two branches, the methyl branch and the carbonyl branch. The methyl branch is a linear metabolic branch that converts CO_2_ to methyl groups through six sequential steps. When acetogens autotrophically grow on CO_2_/H_2_, the first reaction of methyl branch is the reduction of CO_2_ to formate catalyzed by formate dehydrogenase (FDH) using two electrons. Formate then undergoes a reaction with tetrahydrofolate (THF) to form formyl-THF, catalyzed by formyl-THF synthetase (FTS) and consuming one ATP. Subsequently, formyl-THF is converted to methenyl-THF by formyl-THF cyclohydrolase (FTC), leading to the release of one H_2_O. The reduction of methenyl-THF to methylene-THF, and eventually to methyl-THF, occurs through the successive actions of methylene-THF dehydrogenase (MDH) and methylene-THF reductase (MTHFR), with each step involving the consumption of two electrons. In the last step of methyl branch, the methyl group of methyl-THF is transferred to a corrinoid iron-sulfur-containing protein (CoFeSP) via the methyltransferase (MT). The reducing steps of methyl branch require a total of six electrons, while the specific coenzymes carrying the reducing equivalents are diverse and still partially unrevealed (Drake and Daniel, 2004; Schuchmann and Müller, 2016). The carbonyl-branch involves a single reaction facilitated by a multi-component enzyme complex known as carbon monoxide dehydrogenase/acetyl-CoA synthase (CODH/ACS), which catalyzes the reduction of CO_2_ to CO using two electrons. In the final step of WLP, the bi-functional CODH/ACS enzyme complex catalyzes the reaction that converts CO, methyl-CoFeSP and CoA into acetyl-CoA. Certain acetogens like *Moorella thermoacetica* and *Clostridium ljungdahlii* exhibit the capability of thriving using CO as sole carbon and energy source. In this situation, CO_2_ is generated from CO through the catalysis of CODH initially, and then enters the methyl branch (Ragsdale, 2004). Consequently, CO directly binds to the CODH/ACS. Ultimately, acetyl-CoA is phosphorylated to acetyl phosphate and further metabolized to acetate by phosphotransacetylase (PTA) and acetate kinase (ACK), releasing one molecule of ATP.

As described above, the fundamental chemistry of the methyl branch is the same in different acetogenic species. However, the enzymes that catalyze the reduction of CO_2_ to formate and the reduction of methylene-THF to methyl-THF are different, involving different coenzymes. In the step of CO_2_ reduction to formate, the standard redox potential (E_0_ʹ) of the CO_2_/formate couple is −432 mV (Thauer et al., 1977). Therefore, oxidation of NADH (E_0_ʹ = −320 mV) is not sufficient to drive this reaction. Instead, reduced ferredoxin [(ferredoxin (Fd)/reduced ferredoxin (Fd^2−^)) E_0_ʹ = −450 mV], NADPH [(NADP^+^/NADPH) E_0_ʹ = −370 mV], and H_2_ [(H^+^/H_2_) E_0_ʹ = −414 mV] all have physiological redox potentials that can potentially drive the reaction (Schuchmann and Müller, 2014). Different acetogens have evolved to use different electron donors (Figure 1A). In the model organism *Acetobacterium woodii*, a hydrogen-dependent CO_2_ reductase (HDCR) directly employs H_2_ as the electron donor for CO_2_ reduction (Schuchmann and Müller, 2013). The HDCR complex comprises a hydrogenase module (HydA2) and a formate dehydrogenase module (FdhF1 or FdhF2), linked by two electron-transferring subunits (HycB2 and HycB3). In the case of *M. thermoacetica*, the reduction of CO_2_ to formate is catalyzed by a two-subunit NADPH-dependent FDH, despite the unfavorable bioenergetics with NADPH (E_0_ʹ = −370 mV) (Yamamoto et al., 1983; Rosenbaum and Müller, 2023). The genome of *C. ljungdahlii* harbors three gene clusters encoding potential FDHs (Schuchmann and Müller, 2014). The first gene cluster (Clju_c06990–07080), encodes an FDH and an electron-bifurcating hydrogenase, and this complex putatively use either H_2_ or Fd^2-^ and NADPH as electron donors (Wang et al., 2013a; Nagarajan et al., 2013; Mock et al., 2015). The second gene cluster (Clju_c20030–20040) encodes an FDH and a small ferredoxin-like protein, and this complex presumably accepts electrons from Fd^2-^. The third FDH is encoded by Clju_c08930, an isogene of Clju_c20040 (Schuchmann and Müller, 2014).

The MTHFR that catalyzes the reduction of methylene-THF to methyl-THF also exhibits diversity among various acetogens (Figure 1B). The MTHFR complex from *A. woodii* is composed of MetF, MetV, and RnfC2 in a stoichiometry of 1:1:1, with NADH serving as the electron donor (Ragsdale and Ljungdahl, 1984). In *M. thermoacetica*, the genes *metVF* encoding the MTHFR are co-transcribed together with the genes *hdrCBA-mvhD*. The complex MetFV-HdrABC-MvhD is hypothesized to be an electron-bifurcating enzyme, which uses NADH as reductant (Mock et al., 2014). However, the second electron acceptor is unknown yet. The MetF-MetV complex from *C. ljungdahlii* has been purified and characterized, indicating that the enzyme uses Fd^2-^ as the electron donor and is not capable of electron bifurcation (Yi et al., 2021). However, a proposed energy metabolic scheme suggests that this process cannot physiologically occur, as it would result in a negative ATP gain during autotrophic acetogenesis. It is postulated that the MTHFR may assemble into a large, flexible complex involving other electron-bifurcating enzymes (Öppinger et al., 2022).

### 2.2 Energy conservation

**FIGURE 2:**
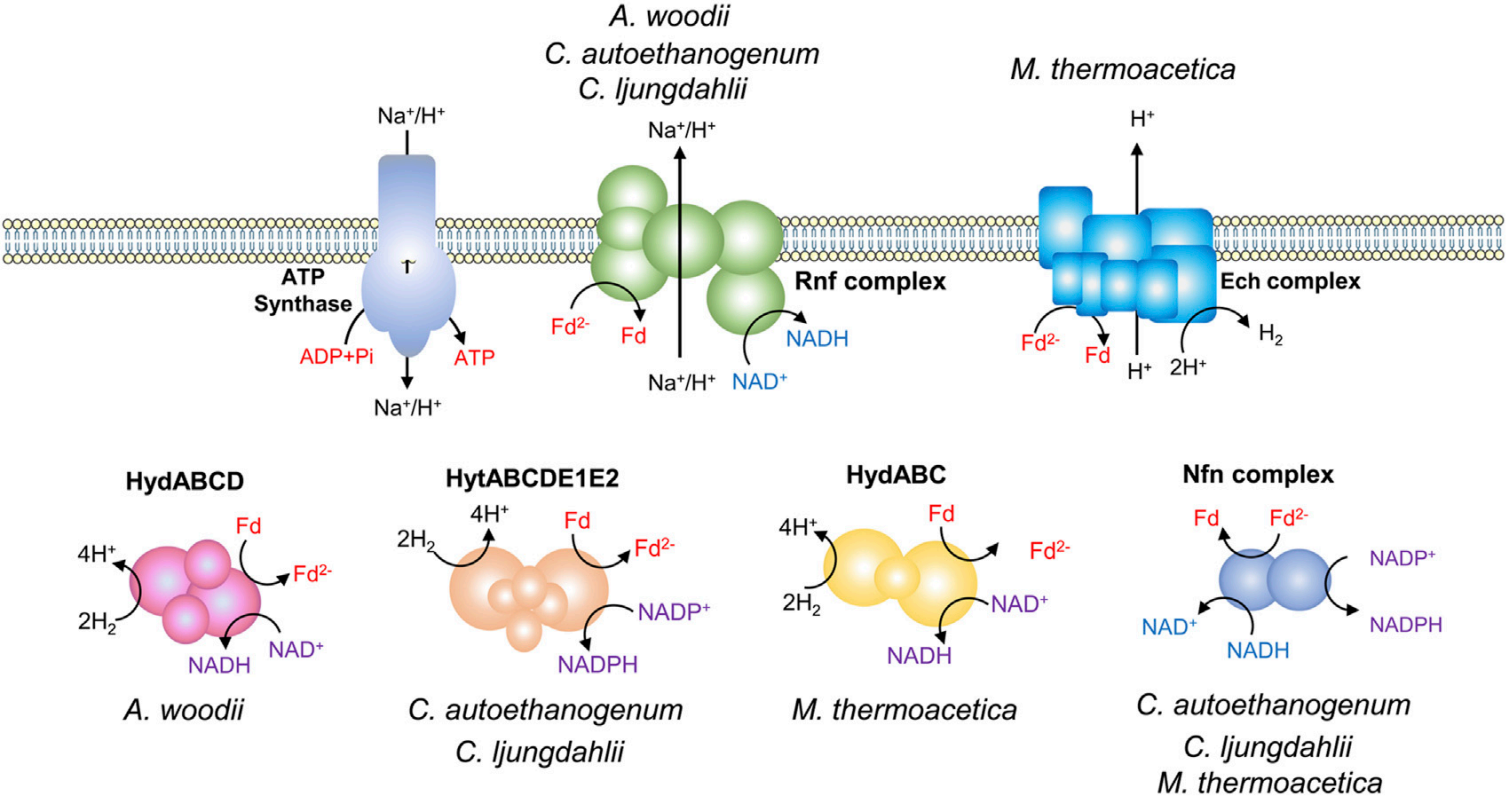
Energy conservation in acetogens. Different electron-bifurcating hydrogenases of acetogens: HydABCD from *A. woodii*, HytABCDE1E2 from *C. autoethanogenum* and *C. ljungdahlii*, and HydABC from *Moorella thermoacetica*. Nfn complex, the electron-bifurcating and ferredoxin-dependent transhydrogenase.

Acetate kinase reaction during acetogenesis synthesizes one ATP via substrate-level phosphorylation (SLP). While, the activation of formate consumes one ATP, resulting in zero net ATP yield through SLP. This highlights a chemiosmotic energy conservation mechanism for ATP synthesis in acetogens, which relies on an Fd^2-^-driven ion pump complex (Figure 2). There are two different ion pump complexes in acetogens, Rnf (Rhodobacter nitrogen fixation) and Ech (Energy-converting hydrogenase) (Buckel and Thauer, 2013). The Rnf complex found in *A. woodii*, *C. ljungdahlii*, and *Clostridium autoethanogenum*, is a ferredoxin:NAD^+^ oxidoreductase, and it pumps Na^+^ or H^+^ outside the cell when transferring electrons from Fd^2-^ to NAD^+^ (Tremblay et al., 2012; Hess et al., 2013; Mock et al., 2015). In contrast, the Ech complex found in *M. thermoacetica* and *Thermoanaerobacter kivui* has ferredoxin:H^+^ oxidoreductase activity and pumps H^+^ (Katsyv and Müller, 2022; Rosenbaum and Müller, 2023). The transmembrane H^+^ or Na^+^ gradient created by Rnf or Ech drives the ATP synthase to generate ATP (Schoelmerich and Müller, 2019). In comparison to *A. woodii*, *C. ljungdahlii*, and *C. autoethanogenum*, *M. thermoacetica* exhibits a notable difference in the presence of cytochromes and quinones, which probably contribute to energy conservation (Gottwald et al., 1975; Rosenbaum and Müller, 2023). *M. thermoacetica* harbors a putative “headless” NADH dehydrogenase that may function as an H^+^ translocating enzyme, driving the ATP synthase by utilizing Fd^2-^ as an electron donor and quinone as an electron acceptor (Pierce et al., 2008). The largest thermodynamic barrier in the WLP is the reduction of CO_2_ to CO, as the redox couple has a very low standard redox potential (E_0_ʹ = −520 mV) (Thauer et al., 1977). Among the identified coenzymes in the WLP, only Fd^2-^ can provide electrons for the reaction (Schuchmann and Müller, 2014).

Every electron for autotrophic acetogenesis from H_2_ and CO_2_ is derived from the oxidation of hydrogen, facilitated by hydrogenases (Figure 2) (Schuchmann and Müller, 2014; Di Leonardo et al., 2022). *A. woodii* has a four-subunit soluble hydrogenase, HydABCD, which contains iron-sulphur centers and flavin (Schuchmann and Müller, 2012). The electron-bifurcating HydABCD catalyzes the oxidation of H_2_, coupling the endergonic reduction of Fd and the exergonic reduction of NAD^+^. Fd and NAD^+^ are reduced simultaneously in a 1/1 stoichiometry (Schuchmann and Müller, 2012). The electron-bifurcating hydrogenases from *C. ljungdahlii* and *C. autoethanogenum* are composed of six subunits (HytABCDE1E2) and are NADP^+^ and Fd-dependent. The hydrogenase forms a tight complex with the formate dehydrogenase FdhA (Wang et al., 2013a; Nagarajan et al., 2013; Mock et al., 2015). The electron-bifurcating hydrogenase of *M. thermoacetica*, HydABC consists of three subunits, which catalyzes the oxidation of hydrogen with NAD^+^ and Fd as electron acceptors (Wang et al., 2013b). Part of the Fd^2−^ is used in the reductive reactions of acetogenesis and the remaining Fd^2−^ can be used by the Rnf or Ech complex to generate a chemiosmotic gradient. In addition, due to the involvement of different cofactors in the energy metabolism, some acetogens such as *C. ljungdahlii*, *C. autoethanogenum*, and *M. thermoacetica* have an electron-bifurcating ferredoxin-dependent transhydrogenase (Nfn complex) (Huang et al., 2012; Nagarajan et al., 2013; Mock et al., 2015). It is capable of producing NADPH from Fd^2−^ and NADH through electron bifurcation, regulating the redox pool.

In recent years, the understanding of acetogenic metabolism has gradually deepened, which supports the metabolic engineering of acetogens in principles. Meanwhile, complete genomic sequences have been published for the species including *A. woodii*, *M. thermoacetica*, *C. ljungdahlii*, *C. autoethanogenum*, and *Clostridium aceticum*, making genetic modification in those acetogens possible.

## 3 Genetic manipulation tools in acetogens

The genetic toolkit available for acetogens has significantly expanded in the past decade. In addition to plasmid-based gene expression, genome editing approaches through ClosTron, homologous recombination, CRISPR-Cas system, and heterologous integration have been developed for acetogens (Joseph et al., 2018).

### 3.1 DNA transfer

An efficient genetic system relies on a reliable DNA transfer method. DNA transfer in most acetogens is limited due to their Gram-positive cell wall (Bourgade et al., 2021). Currently, two usually used transformation methods for introducing foreign DNA into acetogens are electroporation and conjugation. The highly efficient electroporation of acetogens is usually described as treating early-log phase cells with cell-wall-weakening agents such as glycine or DL-threonine and osmotic stabilizers like sucrose; and then collecting and preparing the cells into competent cells; after that, electroporation is performed (Köpke et al., 2010; Kita et al., 2013; Leang et al., 2013; Straub et al., 2014; Shin et al., 2019; Zhao et al., 2019). The transformation efficiency varies depending on the species of the host cell and the type of plasmids, ranging from approximately 10^1^–10^5^ CFU per μg DNA (Zhao et al., 2019; Moreira et al., 2023). The DNA transfer by conjugation involves a donor bacterial strain such as *Escherichia coli* to transfer single-stranded DNA to the acceptor cells through cell-to-cell contact. Conjugation is applied in *C. autoethanogenum* (Mock et al., 2015; Nagaraju et al., 2016) and *Clostridium carboxidivorans* (Cheng et al., 2019). It is worth noting that thermophilic acetogen *T. kivui* naturally took up plasmid DNA in exponential growth phase, with a transformation frequency of up to 3.9 × 10^−6^ (Basen et al., 2018).

The native restriction modification (RM) systems are widespread in acetogens, which is a barrier to acetogen transformation. RM systems consist of restriction endonucleases and corresponding methyltransferases. The endonucleases recognize and cut specific DNA sequences, while the methyltransferases methylate the corresponding sequences in the host genome to protect it from cleavage (Vasu and Nagaraja, 2013). Among the four types of RM systems identified in bacteria, types I, II, and III target and cleave unmethylated DNA, while type IV recognizes DNA with foreign methylation patterns. For hosts with types I, II, or III RM systems, to circumvent the protective systems, plasmids can be methylated prior to transformation. One feasible approach is to express the native methyltransferases of the host in *E. coli* to create a methylation strain (Yasui et al., 2009). An example is the transformation of *M. thermoacetica*, where three native methyltransferases were expressed in *E. coli* to methylate the plasmid in order to counter the type I and type II RM systems of *M. thermoacetica* (Kita et al., 2013). RM systems in acetogens are often species-specific. Updated information of identified restriction endonucleases and methyltransferases is available in REBASE database (Roberts et al., 2023). In addition, conjugation is believed to allow partial evasion of the host restriction-modification barriers (Purdy et al., 2002; Cheng et al., 2019). Some acetogens like *C. autoethanogenum* have type IV RM systems, and plasmids constructed from dam^+^/dcm^+^ *E. coli* strains like DH5α will be recognized and degraded (Woods et al., 2019). To prevent this, plasmids can be constructed in *E. coli* strains with different dam/dcm background. One successful example is the transformation of *Clostridium* sp. AWRP, which has never succeeded in electroporation with dam^+^/dcm^+^ DNA, whereas achieved transformation efficiency of 3.2 × 10^2^ CFU/μg DNA with dam^−^/dcm^−^ DNA (Kwon et al., 2024). In addition to electroporation, the problem is also present in conjugative transfer. When *C. autoethanogenum* was the recipient, the commonly used conjugative donor strain *E. coli* CA434 (dam^+^/dcm^+^) exhibited a conjugation efficiency of 1.05 × 10^−7^ per donor cell. While the donor strain of *E. coli* sExpress which was obtained by introducing R-factor R702 into *E. coli* NEB Express strain (dam^+^/dcm^−^) had an efficiency of 7.94 × 10^−5^ per donor cell (Woods et al., 2019).

### 3.2 Plasmids for metabolic engineering in acetogens

Genetic manipulation of acetogens inevitably involves the introduction of foreign DNA, which requires plasmid vectors. Acetogens are Gram-positive bacteria, whereas recombinant plasmids are typically constructed and amplified in *E. coli* and then “shuttled” into the target acetogens. Therefore, shuttle vectors must have both Gram-positive and Gram-negative replicons. The Gram-positive replicons are often derived from natural Gram-positive bacterial plasmids, such as pBP1 from *Clostridium botulinum* (van Treeck et al., 1981), pCB102 from *Clostridium butyricum* (Collins et al., 1985), pIM13 from *Bacillus subtilis* (Monod et al., 1986), pIP404 from *Clostridium perfringens* (Garnier and Cole, 1988), and pCD6 from *Clostridium difficile* (Minton et al., 2004). Recently, a low-copy native plasmid pCA from *C. autoethanogenum* was characterized and applied in heterologous expression, which may further facilitate physiological study and metabolic engineering of acetogens (Nogle et al., 2022). Table 1 shows a list of the Gram-positive replicons in shuttle plasmids that have been applied in acetogens. In certain cases, plasmids do not require replication and stable existence within the acetogen host. These plasmids, known as suicide vectors, have Gram-negative but not Gram-positive origins. Hence, they can be constructed and amplified in *E. coli* and transformed into acetogens for genomic editing (Tremblay et al., 2012). The Gram-negative replicons that are commonly used in *E. coli*-Acetogen shuttle vectors are ColE1, p15a, and pUC (Heap et al., 2009). In addition, antibiotic resistance genes including *ermB*, *catP*, and *tetA* are most extensively used selection markers for recombinant screening of acetogens. For thermophilic acetogens like *M. thermoacetica*, utilizing an auxotrophic mutant as a screening tool may be an optimal choice (Kita et al., 2013; Basen et al., 2018). In addition, the thermostable kanamycin resistance gene derived from *Streptococcus faecalis* (Trieucuot and Courvalin, 1983) has been used in thermophilic acetogens, including *M. thermoacetica* (Iwasaki et al., 2013) and *T. kivui* (Basen et al., 2018).

**TABLE 1:** List of the Gram-positive replicons applied in acetogens.

Replicons	Species	References
pBP1	*C. autoethanogenum*	Liew et al. (2022), Nogle et al. (2022)
	*C. ljungdahlii*	Ueki et al. (2014)
	*A. woodii*	Hoffmeister et al. (2016)
	*C. carboxidivorans*	Cheng et al. (2019)
	*E. limosum* ^a^	Shin et al. (2019)
pCB102	*C. difficile*	Heap et al. (2010)
	*C. autoethanogenum*	Liew et al. (2016), Liew et al. (2017), Nagaraju et al. (2016)
	*C. ljungdahlii*	Tremblay et al. (2012), Leang et al. (2013), Huang et al. (2016)
	*A. woodii*	Hoffmeister et al. (2016)
	*E. limosum* ^a^	Shin et al. (2019)
pIM13	*C. autoethanogenum*	Köpke et al. (2014)
	*C. ljungdahlii*	Köpke et al. (2010), Philipps et al. (2019)
	*E. limosum* ^a^	Shin et al. (2019)
pIP404	*C. ljungdahlii*	Köpke et al. (2010), Bengelsdorf et al. (2016), Woolston et al. (2018)
	*A. woodii*	Hoffmeister et al. (2016)
	*E. limosum*	Shin et al. (2019)
pCD6	*C. autoethanogenum*	Liew et al. (2017), Annan et al. (2019)
	*C. ljungdahlii*	Annan et al. (2019)
	*A. woodii*	Hoffmeister et al. (2016)
	*E. limosum* ^a^	Shin et al. (2019)
pCA	*C. autoethanogenum*	Nogle et al. (2022)

^a^ Extremely low transformation efficiency.

In efforts to improve the availability of shuttle plasmids tailored for clostridial hosts and specific applications, the pMTL80000 modular plasmid system was designed (Heap et al., 2009). This system facilitates the versatile construction of *Clostridium-E. coli* shuttle plasmids by integrating standardized modules comprising Gram-positive and Gram-negative replicons, selection markers, and other functional modules. The plasmid series has been extensively applied in acetogens, including *Clostridium* species such as *C. autoethanogenum* (Köpke et al., 2014), *C. ljungdahlii* (Ueki et al., 2014)*,* and *C. carboxidivorans* (Cheng et al., 2019), as well as other acetogen species like *A. woodii* and *Eubacterium limosum* (Hoffmeister et al., 2016; Shin et al., 2019).

### 3.3 Genome editing tools applied in acetogens

**FIGURE 3:**
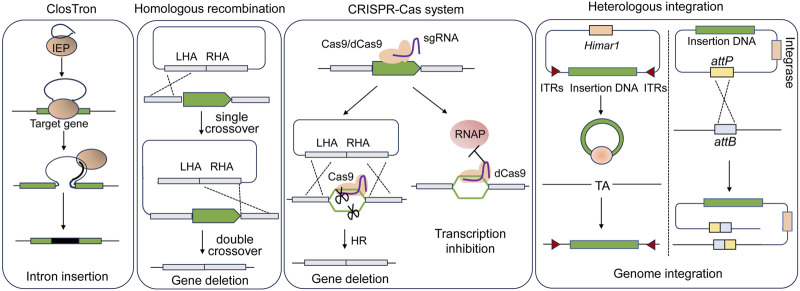
Scheme of genome engineering tools applied in acetogens. LHA, left homologous arm; RHA, right homologous arm; IEP, intron encoded protein; RNAP, RNA polymerase; ITRs, inverted terminal repeats.

It is feasible to introduce genes into the hosts for overexpressing endogenous genes or heterologous metabolic pathways using replicable plasmids. However, maintaining plasmids for gene expressions in the host cells requires the use of antibiotics, which is unfavorable for industrial production. Moreover, there is a concern regarding the potential spread of resistance markers into the environment. In this context, several genome editing tools allowing for gene disruption and fragment insertion in acetogens have been developed (Figure 3; Table 2).

**TABLE 2:** Genetic tools used in acetogens.

Species	Manipulations	References
ClosTron		
*C*. *difficile*	Disruption of *spo0A* involved in spore formation	Heap et al. (2010)
*C. autoethanogenum*	Disruption of (FeFe)- and (NiFe)-hydrogenase genes	Mock et al. (2015)
*C. autoethanogenum*	Disruption of PEP carboxykinase, glyceraldehyde-3-phosphate dehydrogenase, and Nfn complex genes	Marcellin et al. (2016)
*C. autoethanogenum*	Disruption of *cooS1*, *cooS2*, and *acsA* that encoding CO dehydrogenases	Liew et al. (2016)
*C. autoethanogenum*	Disruption of *adhE1*, *adhE2*, *aor1*, and *aor2*	Liew et al. (2017)
*C. ljungdahlii*	Disruption of *adhE1*	Bengelsdorf et al. (2016)
Homologous recombination		
*C. ljungdahlii*	Deletion of *rnfAB* gene of Rnf Complex	Tremblay et al. (2012)
*C. ljungdahlii*	Deletion of *fliA* via double crossover; Deletion of *adhE1* and *adhE2*	Leang et al. (2013)
*C. ljungdahlii*	Insertion of a butyrate production pathway by single crossover; Removel of vector backbone by Cre-Lox system; Disruption of *adhE1* and *ctf* gene by single crossover	Ueki et al. (2014)
*C. autoethanogenum*	Construction of *ΔpyrE* strain; Deletion of *adhE1*, *adhE1+2*, and *aor2*	Liew et al. (2017)
*M. thermoacetica*	Construction of *ΔpyrF* strain; Insertion of lactate dehydrogenase gene	Kita et al. (2013)
*A*. *woodii*	Construction of *ΔpyrE* strain; Deletion of ∼5-kb *rnf* operon	Westphal et al. (2018)
*A*. *woodii*	Construction of *ΔpyrE* strain; Deletion of *lctCDEF* genes for lactate production	Schoelmerich et al. (2018)
*A*. *woodii*	Construction of *ΔpyrE* strain; Deletion of *hydBA* genes	Wiechmann et al. (2020)
*T*. *kivui*	Construction of *ΔpyrE* strain; Deletion of 1-phosphofructosekinase gene	Basen et al. (2018)
CRISPR-Cas		
*C. ljungdahlii*	Constitutively expressed SpCas9 mediated deletion of *pta*, *adhE1*, *ctf*, and *pyrE*	Huang et al. (2016)
*C. ljungdahlii*	Deactivated SpCas9 mediated knockdown of *pta*, *adhE1*, and *aor2*	Woolston et al. (2018)
*C. ljungdahlii*	SpCas9 mediated insertion of *attB* site and elimination of free or integrated plasmid via single-crossover	Huang et al. (2019)
*C. ljungdahlii*	Constitutively expressed FnCas12a mediated deletion of *pyrE*, *pta*, *adhE1*, and *ctf*; ddFnCas12a mediated knockdown of *cooS1*, *fts*, CLJU_c04990, and *adhE1*	Zhao et al. (2019)
*C. ljungdahlii*	C-to-T substitution in *adhE1*, *adhE2*, *aor1*, and *aor2* mediated by dCas9 and activation-induced cytidine deaminase	Xia et al. (2020)
*C. autoethanogenum*	Anhydrotetracycline-induced SpCas9 mediated deletion of *adh* and *2, 3-bdh*	Nagaraju et al. (2016)
*C. autoethanogenum*	Transcriptional repression of *sADH* and *budA* by deactivated Cas9	Fackler et al. (2021a)
*C. autoethanogenum*	Ribo-Cas mediated deletion of *adhE1a*; SIBR-Cas mediated deletion of *pta*	Dykstra et al. (2022)
*C. autoethanogenum*	Endogenous CRISPR/Cas mediated deletion of *pyrE*	Poulalier-Delavelle et al. (2023)
*A*. *woodii*	Endogenous CRISPR/Cas mediated deletion of *pyre*, *pheA*, and *hsdR1*; Knock-in of the FAST reporter gene at the *pheA* locus	
*E*. *limosum*	Anhydrotetracycline-induced SpCas9 mediated deletion of *folD* and *acsC*; Knockdown of genes in the WLP and fructose-PTS system by dCas9	Shin et al. (2019)
Heterologous integration		
*C. ljungdahlii*	Insertion of acetone biosynthesis pathway into TA sites via Himar1 transposase	Philipps et al. (2019)
*C. ljungdahlii*	Site-specific integration of butyrate synthesis pathway mediated by phage serine integrase	Huang et al. (2019)

#### 3.3.1 ClosTron

The ClosTron, a widely applied gene disruption tool in *Clostridium* species, utilizes the group II Ll.LtrB intron derived from *Lactococcus lactis* (Heap et al., 2007; Heap et al., 2010). Facilitated by the reverse transcription activity of the intron-encoded protein LtrA, the intron integrates into the target genomic site through an RNA-mediated retrohoming mechanism (Karberg et al., 2001). To facilitate the selection of the mutants, antibiotic resistance genes are designed as a retrotransposition-activated selectable marker (RAM). Once the intron is inserted, the genes regain activity, enabling the identification of mutants through corresponding antibiotic selection (Zhong et al., 2003). ClosTron has been successfully employed in the study of acetogens, such as *C. difficile* (Heap et al., 2010), *C. autoethanogenum* (Mock et al., 2015; Liew et al., 2016; Marcellin et al., 2016; Liew et al., 2017), and *C. ljungdahlii* (Bengelsdorf et al., 2016). For instance, the three putative CODH genes, *cooS1, cooS2* and *acsA* in *C. autoethanogenum*, were individually disrupted using ClosTron, confirming that only *acsA* is essential for the autotrophic growth (Liew et al., 2016). Despite its convenience in gene disruption, ClosTron does have limitations. The efficiency significantly decreases when the length of the inserted fragment exceeds 1 kb (Plante and Cousineau, 2006). Additionally, ClosTron is not suitable for studying the function of specific genes in a polycistronic gene cluster, as the insertion of the intron simultaneously silences downstream genes in the cluster (Kuehne et al., 2019; Wang et al., 2023).

#### 3.3.2 Homologous recombination

With the improvement of transformation efficiency, gene deletion was demonstrated to be feasible by using suicide plasmids through homologous recombination (HR) in acetogens. Early attempts at HR-based gene deletion were carried out in *C. ljungdahlii*, resulting in successful deletion of the *rnfAB* (Tremblay et al., 2012) and the *adhE1* and *adhE2* genes that encode bi-functional aldehyde/alcohol dehydrogenases (Leang et al., 2013). Furthermore, to streamline mutant screening and allow for reusing selection markers, the counterselection marker *catP* and the Cre-lox system was adopted to eliminate the plasmid backbone (Ueki et al., 2014). Using this system, the butyrate synthesis pathway was integrated into the *C. ljungdahlii* genome. The *pyrE* and *pyrF* genes, essential for pyrimidine synthesis can serve as both positive and negative selection markers. The uracil phosphoribosyltransferase and orotate phosphoribosyltransferase encoded by *pyrE* and *pyrF*, respectively, can convert non-toxic 5-fluoroorotic acid (5-FOA) into toxic 5-fluoro-dUMP. Uracil auxotrophic strains generated by disrupting either *pyrE* or *pyrF* can be screened by the addition of uracil and 5-FOA. The auxotrophic mutant, when complemented with *pyrE* or *pyrF* through plasmids or genome integration, can be easily isolated in uracil-free medium. This approach has been successfully employed not only in mesophilic acetogens (Liew et al., 2017; Schoelmerich et al., 2018; Westphal et al., 2018; Wiechmann et al., 2020) but also in thermophilic acetogens such as *M. thermoacetica* and *T*. *kivui* (Kita et al., 2013; Basen et al., 2018), for which the antibiotics is not always working.

#### 3.3.3 CRISPR-Cas

In recent years, the CRISPR-Cas gene editing system has emerged as an efficient, targeted, and scarless gene editing technique that is widely used. The application of CRISPR-Cas gene knockout systems and their derivatives has been proved successful in acetogens.

The Type II CRISPR-Cas9 system from *Streptococcus pyogenes* is most extensively applied (Cong et al., 2013). The Cas9 nuclease of the system is targeted by a short single guide RNA (sgRNA) and performs a double-stranded cleavage on the target DNA, which introduces a double-stranded break (DSB) on the chromosome. DSBs are repaired through homologous recombination using simultaneously introduced donor DNA as the template that contains complementary sequences to both the upstream and the downstream regions of the DSBs. In *Clostridium* lacking a representative recombination repair system, Cas9 is thought to function through a counterselection mechanism, where cells that undergo gene editing via occasional recombination with donor DNA survive, while unedited cells die from DSBs introduced by Cas9 (Joseph et al., 2018). The CRISPR-Cas9 gene editing tool, which utilizes a constitutive promoter to drive *cas*9 expression, has been successfully applied in *C. ljungdahlii* (Huang et al., 2016). However, conducting transformant screening and mutant screening in a single step has led to a low overall rate of positive outcomes.

In such cases, utilizing inducible promoters to control the expression of *cas9* is a more favorable approach. This approach divides transformant screening and mutant screening into two steps, allowing for the screening process to be conducted in the absence or presence of inducers. This ensures that a sufficient number of colonies can be obtained in each screening process. For example, in *C. autoethanogenum*, a modified anhydrotetracycline-inducible promoter *P*_IPL12_ was applied to control the expression of *cas9*, achieving an editing efficiency of over 50% (Nagaraju et al., 2016). Similarly, the anhydrotetracycline-inducible promoter *P*_tetO1_ was employed to control *cas9* expression in *E. limosum*, resulting in 100% editing efficiency upon induction (Shin et al., 2019). A theophylline-induced riboswitch-based system was able to efficiently control the expression of *cas9* from translational level in several clostridial species (Cañadas et al., 2019). The efficiency of the Ribo-Cas system has been validated in *C. autoethanogenum* (Dykstra et al., 2022).

The Cas12a, also known as Cpf1, from *Francisella novicida* has also been successfully utilized for gene editing in *C. ljungdahlii* (Zhao et al., 2019). Cas12a recognizes a PAM sequence of -TTN- that is suitable for genomes with low GC content. In contrast to Cas9, the CRISPR-Cas12a system is preferred for multi-gene editing due to its requirement of only a single promoter and terminator to express multiple spacers and repeats (Zetsche et al., 2017). Patinios et al. (2021) developed a Self-splicing Intron-Based Riboswitch system to tightly control the expression of FnCas12a (SIBR-Cas) and the system has been employed in C. *autoethanogenum* for gene deletion (Dykstra et al., 2022).

CRISPR-Cas systems offer alternative methods for genetic modification in addition to the conventional double-strand break-homology directed repair (DSB-HR) gene editing process. The binding of deactivated Cas9 (dCas9) or DNase-deficient Cas12a (ddCas12a) to the target sites disrupts the transcription process of RNA polymerase, leading to decreased expression level of the target genes. Referred to as CRISPR interference (CRISPRi), this system has been effectively employed for gene knockdown in acetogens (Woolston et al., 2018; Shin et al., 2019; Zhao et al., 2019; Fackler et al., 2021a). For example, employing ddCas12a for CRISPRi in *C. ljungdahlii* led to a notable decrease of 88%–99% in the expression levels of multiple target genes (Zhao et al., 2019). Targeting the *budA* gene in *C. autoethanogenum* using dCas9 and various gRNAs resulted in a significant 75% reduction in 2,3-butanediol production (Fackler et al., 2021a). Furthermore, Xia et al. (2020) developed the Target-aid CRISPR system using deactivated Cas9 in conjunction with cytidine deaminase to facilitate precise C to T mutations at targeted sites.

Recently, the endogenous Type I-B CRISPR-Cas systems in *A. woodii* and *C. autoethanogenum* have been identified, characterized, and successfully employed for gene knockout in these organisms (Poulalier-Delavelle et al., 2023). The CRISPR-Cas system from *A. woodii*, with a putative PAM sequence of -CCA-, has been used to knockout the *pyrE*, *pheA*, and *hsdR1* genes, as well as for the integration of the reporter gene *FAST* at the *pheA* locus. Similarly, the endogenous CRISPR-Cas system of *C. autoethanogenum*, with a putative PAM sequence of -TCA-, was employed to successfully knockout *pyrE*.

#### 3.3.4 Heterologous integration

With regard to the introduction of large DNA fragments, targeted genomic heterologous integration has been achieved through homologous recombination based on single crossover in *C. ljungdahlii* (Ueki et al., 2014). However, this approach carries the potential risk of a second recombination between the homologous regions that mediate the single-crossover. The CRISPR-Cas system has shown high efficiency in gene deletion in acetogens, yet the insertion of large DNA fragments into the chromosome is challenging due to the low efficiency of homologous recombination in acetogens. Alternatively, heterologous integration can be achieved through non-targeted transposon mutagenesis, wherein transposons carrying foreign DNA are randomly inserted into the host genome. One commonly used transposon is the mariner-type Himar1 derived from *Haematobia irritans*, which is not species-specific and can be used in both prokaryotic and eukaryotic hosts (Lampe et al., 1996; Fu et al., 2008; Cartman and Minton, 2010). The Himar1 transposase recognizes inverted terminal repeats (ITRs) at both ends of the transposons and inserts them randomly into a -TA- target site in the genome. The Himar1 transposase system, controlled by a xylose-inducible promoter, has successfully integrated a 5-kbp acetone synthesis gene cluster into the *C. ljungdahlii* genome, thereby enabling the production of acetone and isopropanol (Philipps et al., 2019).

The attachment/integration (Att/Int) system, facilitated by phage serine integrases, enables precise DNA rearrangement through the phage attachment (*attP*) and bacterial attachment (*attB*) sites in the chromosome, which is a powerful tool for rapid chromosomal integration of large DNA fragments. A phage serine integrase-mediated site-specific heterologous integration system has been implemented in *C. ljungdahlii* (Huang et al., 2019). Initially, the *attB* site from bacteriophage ФCD27 was inserted into the genome using CRISPR-Cas9. Subsequently, in the modified strain, an exogenous plasmid containing the ФCD27 integrase and the *attP* site was integrated at the *attB* site. The effectiveness of this system was further validated through the integration of a 9.1-kbp exogenous butyrate synthesis pathway.

## 4 Achievements of metabolic engineering in acetogens

**FIGURE 4:**
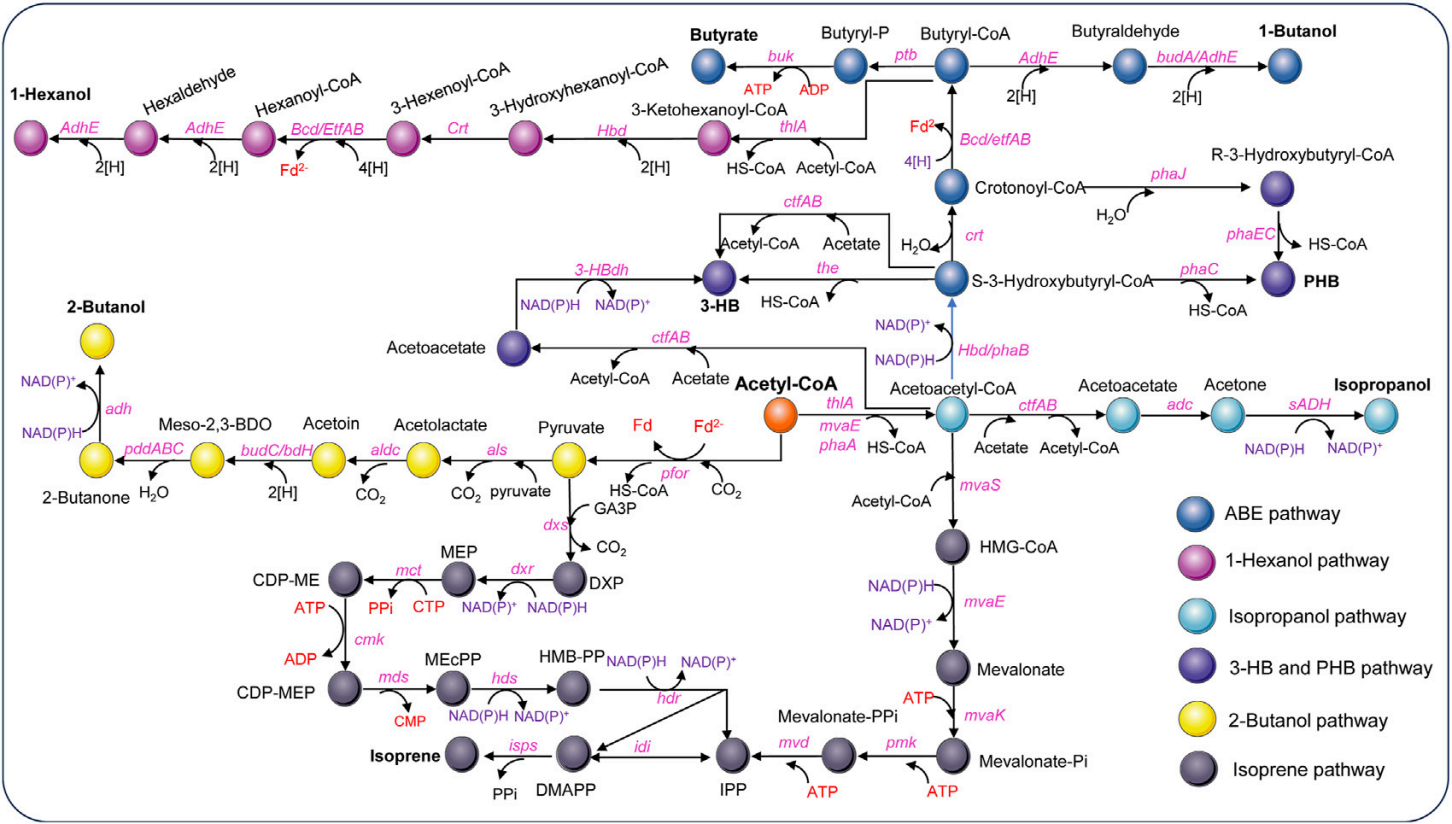
Metabolic pathways for non-natural product synthesis using acetyl-CoA as the starting point in acetogens [Modified from Jin et al. (2020)]. *Adc*, acetoacetate decarboxylase gene; *adhE*, aldehyde/alcohol dehydrogenase gene; *adh*, alcohol dehydrogenase gene; *aldc*, alpha-acetolactate decarboxylase gene; *als*, acetolactate synthase gene; *bcd*, butyryl-CoA-dehydrogenase gene; *bdhA*, butanol dehydrogenase; *buk*, butyrate kinase gene; *budC/bdH*, meso-2,3-butanediol dehydrogenase gene; *cmk*, CDP-ME kinase gene; *crt*, crotonase gene; *ctfAB*, CoA transferase A and B gene; *dxs*, DXP synthase gene; *dxr*, DXP reductoisomerase gene; *etfAB*, genes encoding electron transferring protein A&B; *hbd*, 3-hydroxybutyryl-CoA dehydrogenase gene; *3-HBdh*, 3-HB dehydrogenase gene; *hdr*, HMB-PP reductase gene; *hds*, HMB-PP synthase gene; *idi*, isopentenyl pyrophosphate isomerase gene; *isps*, isoprene synthase gene; *mct*, MEP cytidylyltransferase gene; *mds*, MEcPP synthase gene; *mvaE*, acetyl-CoA acetyltransferase/HMG-CoA reductase gene; *mvaK*, mevalonate kinase gene; *mvaS*, HMG-CoA synthase gene; *mvd*, mevalonate diphosphate decarboxylase gene; *pddABC*, diol/glycerol dehydratase gene; *pfor*, pyruvate ferredoxin oxidoreductase; *phaA*, 3-ketothiolase; *phaB*, NAD(P)H-dependent acetoacetyl-CoA reductase gene; *phaC/phaEC*, encoding polyhydroxyalkanoate synthase; *phaJ*, (R)-enoyl-CoA hydratase gene; *pmk*, phosphomevalonate kinase gene; *ptb*, phosphotransbutylase gene; *sADH*, primary-secondary alcohol dehydrogenase gene; *the*, thioesterase gene; *thlA*, acetyl-CoA acetyltransferase/thiolase gene; Metabolites: HMG-CoA, hydroxymethylglutaryl-CoA; DXP, 1-deoxy-D-xylulose 5-phosphate; MEP, 2-C-methyl-D-erythritol 4-phosphate; CDP-ME, 4-(cytidine 5′-diphospho)-2-C-methyl-D-erythritol; CDP-MEP, CDP-ME2-phosphate; MEcPP, 2-C-methyl-D-erythritol 2,4-cyclodiphosphate; HMB-PP, 4-hydroxy-3-methylbut-2-enyl diphosphate.

The continuous advancement of genetic tools promotes the use of metabolic engineering approaches to accelerate the development of acetogens as cell factories. Extensive efforts have been dedicated to enhancing both C1 fixation efficiency and the production of native biochemicals. The improvement of C1 fixation efficiency is primarily achieved either through the overexpression of specific enzymes in the Wood-Ljungdahl pathway (WLP) such as the selective overexpression of the four THF-dependent enzymes for the processing of formate in *A woodii* (Straub et al., 2014) or the introduction of alternative carbon-fixation pathways such as the glycine synthase-reductase pathway into *E. limosum* (Song et al., 2020). It should be noted that these results were obtained through batch fermentation, which may perform differently under continuous cultivation conditions (Pavan et al., 2022). On the other hand, the enhancement of natural product synthesis mainly revolves around the production of acetate, ethanol, and 2,3-butanediol. This was achieved by overexpressing the enzymes involved in the synthesis pathways of the desired products or by disrupting competing pathways (Banerjee et al., 2014; Straub et al., 2014; Liew et al., 2017). Furthermore, acetogens have been explored as a chassis for the production of non-natural biochemicals by introducing exogenous genes, significantly expanding their product range to include dozens of biochemicals (Figure 4; Table 3) (Fackler et al., 2021b). Notably, several of these products have demonstrated industrially relevant performance levels, with production rates in the range of g/L/h and titers at the tens of g/L level. This section will focus on the exploration of producing non-natural products through metabolic engineering of acetogens.

**TABLE 3:** Non-natural biochemical production using the engineered acetogens.

Species	Target product	Substrates	Levels	References
*C. autoethanogenum*	Acetone; isopropanol	syngas	3 g/L/h	Liew et al. (2022)
	3-HB	syngas	14.63 g/L	Karim et al. (2020)
	PHB	syngas	0.027 g/L, 5.6% per CDW	De Souza Pinto Lemgruber et al. (2019)
	butanol	CO	1.54 g/L	Köpke and Liew (2011)
	hexanol	syngas	0.26 g/L	Vögeli et al. (2022)
	MEK 2-butanol	steel mill gas	Not quantified	Mueller et al. (2018)
*C. ljungdahlii*	Acetone	CO or syngas	∼0.8 g/L; 0.035 g/L	Banerjee et al. (2014), Bengelsdorf et al. (2016), Philipps et al. (2019)
	3-HB	syngas	9.2 g/L; 0.083 g/L/h	Lo et al. (2022)
	PHB	syngas	1.2% per CDW	Flüchter et al. (2019)
	Isopropanol 3-HB	syngas	13.4 g/L; 3.0 g/L	Jia et al. (2021)
	butyrate	CO_2_+H_2_	∼1.6 g/L; 1.01 g/L	Ueki et al. (2014), Huang et al. (2019)
	butanol	syngas	0.15 g/L	Köpke et al. (2010)
	butanol hexanol	CO_2_+H_2_	0.451 g/L; 0.393 g/L	Lauer et al. (2022)
	mevalonate isoprene	syngas	0.068 g/L; 1.5 μg/L	Diner et al. (2017)
*C. coskatii*	3-HB PHB	syngas	0.1 g/L; 1.2% per CDW	Flüchter et al. (2019)
*A. woodii*	Acetone	CO_2_+H_2_	3.02 g/L; 26.4 mg/L/h	Hoffmeister et al. (2016)
	Acetone isopropanol	CO_2_+H_2_	0.44 g/L; 0.87 g/L	Arslan et al. (2022)
	isopropanol	CO_2_+H_2_	0.83 g/L	Höfele et al. (2023)
*M. thermoacetica*	Acetone	CO + H_2_	0.09 g/g-dry cell/h	Kato et al. (2021)

### 4.1 Acetone and isopropanol

Acetone, an important commodity chemical and feedstock for poly (methyl methacrylate) production can be biosynthesized by ABE fermentation. The metabolic pathway involved has been well elucidated (Jones and Woods, 1986). Initially, two molecules of acetyl-CoA are converted to acetoacetyl-CoA by a thiolase (ThlA), and then CoA is transferred to acetate by a CoA transferase (CtfAB) to generate acetoacetate and another acetyl-CoA, and finally acetoacetate is decarboxylated by an acetoacetate decarboxylase (Adc) to generate acetone. However, ABE fermentation typically utilizes sugar as a substrate, leading to growing interest in C1 gas fermentation by acetogens. Acetone is not a natural product of any known acetogens. Proof-of-concept production of acetone has been achieved by plasmid-based heterologous expression of acetone synthesis pathways from *Clostridium acetobutylicum* in several acetogens, such as *C. aceticum* (Schiel-Bengelsdorf and Dürre, 2012), *C. ljungdahlii* (Banerjee et al., 2014), and *A. woodii* (Hoffmeister et al., 2016; Arslan et al., 2022). Studies have shown that acetone can be reduced to isopropanol by an endogenous primary-secondary alcohol dehydrogenase (sADH) in *C. autoethanogenum* (Köpke et al., 2014) and *C. ljungdahlii* (Bengelsdorf et al., 2016). The integration of acetone synthesis pathway into the genome of *C. ljungdahlii* resulted in the production of 0.6 mM acetone and 2.4 mM isopropanol by the engineered strain (Philipps et al., 2019). Instead, Jia et al. (2021) introduced an artificial isopropanol-producing pathway via an expression plasmid incorporating thiolase, CtfAB, Adc, and sADH from *Clostridium* species into *C. ljungdahlii.* The best-performing recombinant strain was able to produce 13.4 g/L of isopropanol in continuous gas fermentation. In addition, plasmid-based heterologous expression of the genes *thlA-ctfAB-adc-sadh* in *A. woodii* led to a maximum isopropanol production of 13.87 mM using CO_2_ and H_2_ as the carbon and energy source (Höfele et al., 2023). Recent advancements by LanzaTech have demonstrated commercial-ready levels of acetone and isopropanol production using genomically integrated strains, with continuous production rates reaching up to ∼3 g/L/h (Liew et al., 2022). Life cycle analysis confirmed a negative carbon footprint for their products. Additionally, Kato et al. successfully integrated a thermophilic acetone production pathway into the genome of *M. thermoacetica*, resulting in acetone yields of 0.04–0.09 g acetone/g-dry cell/h, making a notable contribution to the field of metabolic engineering of thermophilic acetogens (Kato et al., 2021).

### 4.2 3-HB and PHB

3-Hydroxybutyrate (3-HB) is a chiral bioproduct of interest with diverse applications in the synthesis of fine chemicals, pharmaceuticals, biofuels, and bioplastics, particularly polyhydroxybutyrate (PHB) (Tokiwa and Ugwu, 2007). 3-HB can be synthesized from acetyl-CoA through a variety of pathways. There are two main pathways proposed to generate 3-HB in acetogens (Figure 4). Both pathways involve the action of a thiolase to combine two acetyl-CoA molecules, producing acetoacetyl-CoA. However, they differ in subsequent steps. In the Hbd pathway, (S)-3-hydroxybutyryl-CoA dehydrogenase (Hbd) reduces acetoacetyl-CoA to (S)-3-hydroxybutyryl-CoA, which is then converted to 3-HB by a thioesterase catalyzed CoA removal. In contrast, the CtfAB/3-HBdh pathway initially utilizes CtfAB to convert acetoacetyl-CoA to acetoacetate by CoA transfer to acetate. Acetoacetate is then reduced by the 3-HB dehydrogenase (3-HBdh), resulting in the formation of 3-HB. For the heterologous production of 3-HB, a novel CtfAB/3-HBdh pathway consisting of the genes *thlA* and *ctfA/B* from *C. acetobutylicum*, as well as *bdhA* encoding a 3-HBdh from *Clostridioides difficile* 630 was introduced into *C. coskatii* and *C. ljungdahlii* through an expression plasmid (Flüchter et al., 2019). The engineered strain of *C. coskatii* autotrophically grown on syngas produced 0.1 g/L of 3-HB. The recombinant *C. ljungdahlii* strain, however, did not produce any detectable amounts of 3-HB neither under heterotrophic nor under autotrophic growth conditions. Jones et al. (2016) proposed that acetoacetate can undergo native conversion to 3-HB in *C. ljungdahlii*, as their acetone-generating strain, containing all the necessary genes for the CtfAB/3HBdh pathway except for 3-HBdh itself, produced significant levels of 3-HB. Subsequently, an endogenous 3-HB dehydrogenase (3-HBdh) was identified in *C. ljungdahlii*, which could convert acetoacetate to 3-HB in an engineered strain for isopropanol production (Jia et al., 2021). Furthermore, the introduction of the *bdhA* gene from *C*. *difficile* 630 via an expression plasmid into the isopropanol-producing strain facilitated the efficient co-production of isopropanol, 3-HB, and ethanol, resulting in a 3-HB titer of 3 g/L in continuous gas fermentations (Jia et al., 2021). Through *in vitro* prototyping and rapid biosynthetic enzyme optimization from *Clostridium*, the plasmid-based expression of an Hbd pathway in *C. autoethanogenum* demonstrated the highest performance in 3-HB production (Karim et al., 2020). In optimized fermentations, the titer of 3-HB reached 14.6 g/L, with production rates exceeding 1.5 g L^−1^ h^−1^ in a continuous system. Recently, Lo et al. (2022) identified a native (S)-3-hydroxybutyryl-CoA dehydrogenase, Hbd2 in *C. ljungdahlii*. In conjunction with the integration of heterologous thiolase gene, *atoB* from *E. coli* and *ctfAB* from *C. acetobutylicum* into the *pyrE* locus, plasmid based overexpression of both the native *hbd2* and *thl2* from *Clostridium kluyveri* significantly improved yields of 3-HB on syngas, resulting in a titer of 9.2 g/L (Lo et al., 2022).

Poly-3-hydroxybutyrate (PHB) is a biodegradable plastic naturally produced by *Cupriavidus necator* and microbes from *Alcaligenes*, *Azotobacter*, *Bacillus*, *Rhizobium*, and *Pseudomonas* (Bhagowati et al., 2015; Jin et al., 2020). PHB is synthesized through the polymerization of 3-hydroxybutyryl-CoA by PHA synthase. For the production of PHB, the PHB synthesis pathway from *C. necator* was expressed in *C. autoethanogenum* under control of the native WLP promoter. Through bioprocess optimization, a PHB yield of 5.6% per CDW with a titer of 0.027 g/L was obtained on syngas in steady-state chemostat cultures (dilution rate of 1 day^−1^) (De Souza Pinto Lemgruber et al., 2019). In addition, as a proof of concept, production of PHB was also achieved using recombinant *C. coskatii* and *C. ljungdahlii* strain expressing a Clostridial PHB synthesis pathway comprising the genes *thlA*, *hbd*, *crt*, *phaJ*, and *phaEC* (Flüchter et al., 2019) (Figure 4). The recombinant strains of both *C. coskatii* and *C. ljungdahlii* synthesized autotrophically 1.2% PHB per CDW.

### 4.3 Butyrate and butanol

Butyrate and butanol are also non-natural products that cannot be produced by wild-type *C. ljungdahlii* and *C. autoethanogenum*. In order to redirect carbon and electron flow to the production of butyrate, a fragment containing genes of *thlA*, *hbd*, *crt*, *bcd*, *etfAB*, *ptb*, and *buk* from *C. acetobutylicum* was integrated into the putative *pta* promoter region of *C. ljungdahlii*. Meanwhile, PTA-dependent acetate synthesis and a gene (Clju_c39430) encoding a homolog of CoA transferases, were disrupted. The final recombinant strain produced approximately 1.6 g/L of butyrate on CO/CO_2_ (Ueki et al., 2014). In addition, to validate the efficiency of the phage serine integrase-mediated genome engineering, a butyrate production pathway from *C. acetobutylicum* was integrated into *C. ljungdahlii*, enabling the production of 1.01 g/L of butyrate by fermenting syngas (Huang et al., 2019).

The production of butanol by engineered acetogens presents challenges due to requirement of additional reducing equivalents for the conversion of butyryl-CoA into butanol. In a demonstration of principle, a plasmid containing most of the *C. acetobutylicum* butanol synthesis pathway, encompassing *thlA*, *hbd*, *crt*, *bcd*, *adhE*, and *bdhA* genes (Figure 4) under control of the phosphotransbutyrylase operon promoter *P*_ptb_ was introduced into *C. ljungdahlii* and a maximum amount of about 0.15 g/L butanol was detected in batch cultures of the engineered strain during mid-growth phase (Köpke et al., 2010). Another approach was carried out in *C. autoethanogenum*, which also expressed the C4 pathway of *C. acetobutylicum* including *thlA*, *hbd*, *crt*, *bcd*, *etfAB* genes (Köpke and Liew, 2011). The highest butanol production measured in the recombinant strain culture grown on CO was 1.54 g/L. In a recent study, an artificial biosynthesis pathway for butanol and hexanol was developed, incorporating 13 genes from *C. kluyveri* and *C. acetobutylicum* and integrated into the genome of *C. ljungdahlii*. In 2-L fermentations with CO_2_ and H_2_ continuous supply, the recombinant strain produced 451 mg/L of butanol, 122 mg/L of hexanol, as well as 199 mg/L of butyrate and 46 mg/L of caproate (Lauer et al., 2022). Vögeli et al. (2022) optimized and implemented the reverse β-oxidation pathway for the synthesis of C4-C6 acids and alcohols. Then engineered *C. autoethanogenum* strain was able to produce 1-hexanol from syngas, achieving a titer of 0.26 g/L in a 1.5 L continuous fermentation.

When utilizing 2,3-butanediol (2,3-BDO) as the substrate, the biosynthesis of 2-butanol involves two reactions: meso-2,3-BDO is converted to 2-butanone (MEK) by propanediol/glycerol dehydratase, and further reduced to 2-butanol by alcohol dehydrogenase (Adh) using NADPH. Acetogens such as *C. ljungdahlii* and *C. autoethanogenum* are capable of producing 2,3-BDO. However, the production of MEK and 2-butanol requires (R,S)-2,3-butanediol, not (R,R)-2,3-butanediol. Nevertheless, through the use of recombinant microorganisms adapted to overexpress crucial enzymes from *Klebsiella* species in the MEK and 2-butanol biosynthesis pathways, the production of MEK and 2-butanol was presumptively achieved (Mueller et al., 2018).

### 4.4 Isoprene

Isoprene is a hemiterpene (C5H8), used primarily in the production of cis-1,4-polyisoprene, the main component of natural rubber, and is also used in the creation of styrene-isoprene-styrene block copolymers, commonly employed in thermoplastic rubber and adhesives. Isopentenyl-pyrophosphate (IPP) and dimethylallyl pyrophosphate (DMAPP) are the initial precursors for all terpenoid biosynthesis, including isoprene (Schrader and Bohlmann, 2015). There are two distinct pathways existing for the generation of IPP and DMAPP: the 2-C-methyl-D-erythritol 4-phosphate (MEP) pathway, prevalent in most prokaryotes and starts with the condensation of pyruvate and glyceraldehyde-3-phosphate (GA3P), and the mevalonic acid (MVA) pathways, found in most eukaryotes, archaea, and some bacteria, which generally starts with the condensation of two acetyl-CoA molecules (Schrader and Bohlmann, 2015). In an effort to enable isoprene production from syngas, the MVA pathway was introduced into *C. ljungdahlii*, along with the overexpression of IPP isomerase and isoprene synthase (ipsS). However, the titer of isoprene during syngas fermentation only reached approximately 1.5 μg/L (Diner et al., 2017). Regrettably, the process of synthesizing isoprene from C1 gas is highly energy-intensive and requires additional ATP, which poses a significant challenge for acetogens with limited capacity for ATP generation. Therefore, efficient synthesis of isoprene is only possible by coupling it with other energy-generating pathways.

## 5 Strategies to overcome challenges in metabolic engineering of acetogens

### 5.1 Strategies to overcome energy limitations in acetogens

As mentioned above, efforts to genetically modify acetogens for the production of non-natural products have yielded only modest success, with low production efficiency that does not meet the demands of commercial scale production, except a few cases. This may be attributed to insufficient energy supply at autotrophic conditions, as acetogens thrive at the thermodynamic limit of life, and their ATP generation process is inherently impeded (Schuchmann and Müller, 2014). As a result, exogenous metabolic pathway modules introduced through genetic modification can only function sub-optimally under such energy constraints.

#### 5.1.1 Improving the energetic efficiency of alcohol production through AOR route

One way to solve this problem is using an energetic advantage pathway (Charubin et al., 2018). *A. woodii* does not produce ethanol when growing on H_2_ and CO_2_. However, when growing on glucose, it can synthesize ethanol in addition to acetic acid, indicating the presence of metabolic pathway from acetyl-CoA to ethanol. The energy metabolism model of *A. woodii* gives an explanation that during acetogenesis on H_2_ and CO_2_, the net ATP gain was 0.3 (moles of ATP per mole of acetic acid). However, if the metabolic pathway is expanded to include ethanol formation from acetyl-CoA, the ATP gain (moles of ATP per mole of ethanol) in *A. woodii* would be negative (Mock et al., 2015). In contrast, the acetogens of *Clostridia* such as *C. autoethanogenum*, *C. ljungdahlii*, and *C. ragsdalei*, produce ethanol in addition to acetic acid when growing on H_2_ and CO_2_, due to an energy saving pathway for ethanol formation. These acetogens have two possible routes for ethanol formation (Liu et al., 2020; Zhu et al., 2020). The first route involves direct reduction of acetyl-CoA to ethanol by the bifunctional aldehyde/alcohol dehydrogenase (AdhE), with acetaldehyde as an intermediate and NAD(P)H as the reducing agent. In this route, no ATP is produced through SLP. In the second route, acetate is produced first with ATP generation through SLP, followed by its reduction to acetaldehyde by the aldehyde:ferredoxin oxidoreductases (AOR) with Fd^2−^. Acetaldehyde is then reduced to ethanol with NAD(P)H. Compared with the AdhE pathway, the AOR pathway generates more ATP, which is more conducive to ethanol production (Liew et al., 2017). More interestingly, AORs demonstrate less substrate specificity, as experiments reveal their ability to reduce various organic acids such as propionic acid, n-butyric acid, isobutyric acid, and n-caproic acid to their corresponding aldehydes, which suggests that the energy-saving strategy via AOR can be used in the synthesis of a variety of non-natural products in acetogens, such as butanol (Perez et al., 2013; Nissen and Basen, 2019).

#### 5.1.2 Bioprocess combination

Another strategy is to use consortia of different organisms (Molitor et al., 2017; Charubin et al., 2018; Katsyv and Müller, 2020). The autotrophic products of acetogens are primarily the C2 compounds acetate and ethanol, which are the substrates for chain elongation via the reverse β-oxidation pathway (Angenent et al., 2016). Through the coupling of syngas fermentation with the chain elongation bioprocess using *C. kluyver*i (the most widely studied chain-elongating microorganism), C1 gas (CO, CO_2_) has been converted into n-butyrate (C4), n-caproic acid (C6), and even n-caprylic acid (C8) (Diender et al., 2016; Richter et al., 2016; Fernández-Blanco et al., 2022). For instance, a co-culture of *C. aceticum* and *C. kluyveri* at near neutral pH were carried out with continuous syngas supply and maximum concentrations of n-butyrate and n-caproate of 7.0 and 8.2 g/L, respectively, have been yielded (Fernández-Blanco et al., 2022). However, one of the major challenges for this technology is the pH mismatch between the two types of microorganisms (Ganigué et al., 2016). Therefore, the two-step bioprocess in two different reactors is an alternative route to overcome energetic limitations (Gildemyn et al., 2017) and has achieved the production of more energy-dense products such as lipids through the integrated conversion of *M. thermoacetica* and *Yarrowia lipolytica* (Hu et al., 2016), malic acid through the combined bioprocess of *C. ljungdahlii* and *Aspergilus oryzae* (Oswald et al., 2016), and polyhydroxyalkanoates through bioconversion of *C. autoethanogenum* and a highly enriched PHA-accumulating biomass (Lagoa-Costa et al., 2017). Recently, recombinant production of *Pseudomonas aeruginosa* rhamnolipids in *Pseudomonas putida* KT2440 was achieved on *A*. *woodii* cultures grown chemo-autotrophically with CO_2_ and H_2_ (Widberger et al., 2024).

#### 5.1.3 Renewable reducing power supply

As the reduction of CO_2_ with H_2_ or CO as reductants is energetically limited, a potential strategy to overcome the energy limitations in acetogens is the direct supply of electrons by renewable reducing power, such as electricity (Lee et al., 2022). The process that microbes utilize electrons from an electrode to convert CO_2_ into multi-carbon products is known as microbial electrosynthesis (MES). Acetogens such as *C aceticum, M. thermoacetica*, and *C. ljungdahlii*, have been reported to perform MES via extracellular electron transfer (Nevin et al., 2011). Recently, Zhang et al. (2023) reported microbial electrosynthesis of acetate from CO_2_ using an enrichment of saline homoacetogens from Atlantic North Sea sediments under hypersaline conditions. However, it is still poorly verified if the electrons are channeled into the cell via membrane-associated redox components, nanowires, or diffusible electron shuttles like H_2_, flavins, or quinones. For example, extracellular electron supply induced a significant metabolic shift of *C. autoethanogenum* from acetate to lactate and 2,3-butanediol fermentation on fructose. Electron mediators such as methyl viologen or neutral red are necessary to facilitate electron transfer from the electrode to the cells (Kracke et al., 2016). *C. carboxidivorans* showed electricity-enhanced fermentation with higher alcohol production and carbon efficiency compared to open-circuit fermentation. Electron mediators such as methyl viologen and biochar facilitated electricity-driven autotrophic CO_2_ fixation and increased carbon efficiencies in electricity-enhanced mixotrophic fermentations (Cheng et al., 2022). *C. ljungdahlii* was initially reported to be able to perform direct extracellular electron transfer but later studies suggested H_2_ mediation is the likely mode of electron uptake (Boto et al., 2023). Hence, more research is needed to explore the underlying mechanism of extracellular electron transfer.

In addition, approaches such as using C1 liquid feedstocks (Cotton et al., 2020; Flaiz et al., 2021; Litty and Müller, 2021; Neuendorf et al., 2021) and mixotrophy (Jones et al., 2016; Maru et al., 2018; Cheng et al., 2019) are also considered to be promising strategies to overcome energy limitations in acetogens (Lee et al., 2022). However, not all the acetogens can utilize methanol or formate. Meanwhile, methanol or formate tolerance is an issue to be addressed. For sugar co-feeding with gas substrates, carbon catabolite repression (CCR) of the WL pathway may be a problem. Possible catabolite repression or native regulatory mechanisms in mixotrophy should be better studied (Hong and Zeng, 2022).

### 5.2 Strategies for efficient optimization of metabolic flux

The synthesis of some non-natural products is thermodynamically favorable for acetogens, such as butyrate, acetone, and isopropanol. However, the production efficiency was still low in some earlier studies, which was caused by the limited metabolic flow.

#### 5.2.1 Genome-scale metabolic modeling

The systematic description of carbon and energy metabolism of acetogens is not only the basis for our understanding of this ancient metabolic mode, but also an important tool to guide their engineering strategy.

The generation of high-yielding strains hinges on the precise combination of enzyme species involved in the metabolic pathway and their optimal expression. *In-silico* assays such as genome-scale metabolic models (GEMs) are valuable for the initial analysis and estimation of pathway yield and feasibility. For example, a genome-scale model of *C. autoethanogenum* was used to explore alternative ATP-generating pathways. Subsequent *in silico* simulations identified an arginine deiminase pathway for ATP production (Valgepea et al., 2017). The OptKnock can identify gene knockout strategies that are expected to enhance the synthesis of target products (Burgard et al., 2003). Chen and Henson performed *in silico* metabolic engineering studies using a genome-scale reconstruction of *C. ljungdahlii* metabolism combined with the OptKnock computational framework to identify gene knockouts that were predicted to enhance the synthesis of these native products and non-native products, introduced through insertion of the necessary heterologous pathways (Chen and Henson, 2016). Benito-Vaquerizo et al. reported a genome-scale, constraint-based metabolic model to describe growth of a co-culture of *C. autoethanogenum* and *C. kluyveri* on syngas. Community flux balance analysis was used to gain insight into the metabolism of the two strains, their interactions, and to uncover potential strategies for producing butyrate and hexanoate (Benito-Vaquerizo et al., 2020).

#### 5.2.2 Cell-free systems

GEMs may sometimes misjudge pathway feasibility, probably due to the lack of experimental data integration. While *in vivo* pathway optimization with a design-build-test approach is a time-consuming and labor-intensive process. Cell-free systems, thus, can be beneficial for pathway prototyping and rapid optimization. A high-yielding batch cell-free expression (CFE) platform was developed and optimized using *C. autoethanogenum* (Krüger et al., 2020). It allows for the direct application of both circular and linear DNA templates to the CFE reaction for programmable protein synthesis. The CFE platform is expected to not only enhance the protein synthesis toolkit for synthetic biology but also facilitate the rapid screening and prototyping of gene regulatory elements in non-model, industrially relevant microbes. Karim et al. built a platform for *in vitro* prototyping and rapid optimization of biosynthetic enzymes (iPROBE) for cell design. Cell lysates of *E. coli* are enriched with biosynthetic enzymes by cell-free protein synthesis and then metabolic pathways are assembled in a mix-and-match fashion to assess performance. By screening 54 different cell-free pathways for 3-HB production and optimizing a six-step butanol pathway across 205 permutations, a strong correlation (r = 0.79) between cell-free and *C. autoethanogenum* cellular performance was observed. The best-performing pathway was scaled up, leading to an improvement in *in vivo* 3-HB production in *Clostridium* by 20-fold to 14.63 g/L (Karim et al., 2020). The iPROBE was also applied for optimization of acetone and isopropanol metabolic flux in *C. autoethanogenum* (Liew et al., 2022). To increase flux to acetone and eliminate unwanted byproducts, effector genes that cause 3-HB formation were firstly identified by iPROBE and then successively knocked out. Furthermore, iPROBE was used to demonstrate the strong dependence of acetone titer on the concentration of CtfAB by varying levels of the different acetone pathway enzymes. CtfAB was titrated at concentrations ranging from 0.05 to 1.50 μM with Thl and Adc constant at 0.5 μM, increasing acetone titer from the limit of detection to 74.6 mM. These recent results indicate that cell-free systems have become an important technique for metabolic engineering efforts in acetogens.

## 6 Conclusion and perspectives

In the current context of striving for carbon neutrality, acetogens hold significant potential for the sustainable production of fuels and chemicals. Their WLP enables them to efficiently convert CO_2_ into valuable products through fermentations, thereby mitigating carbon emissions and offering environmental benefits. The integration of synthetic biology and metabolic pathway engineering further enhances the industrial applications of acetogens. Currently, there is rapid progress in the synthetic biology and metabolic engineering of acetogens, with various metabolic components and genetic tools successfully employed across different acetogen species. Notably, CRISPR-Cas-based genetic modification techniques have been effectively utilized in several acetogen species, facilitating precise and scarless genome editing, and streamlining strain modification processes. By leveraging advanced genetic modification approaches, the incorporation of heterologous metabolic pathways has enabled the engineered acetogen strains to synthesize diverse non-natural products. Some products, such as acetone and isopropanol, have already achieved industrial-scale production, while others are undergoing laboratory research or feasibility assessments. An inherent challenge in utilizing acetogens for non-natural product synthesis lies in their limited energy gain. Strategies involving energy-efficient pathways, bioprocess integration, and microbial electrosynthesis, are being explored to address this constraint. Although notable advancements have been made in this area, continuous innovation in genetic tools is still needed for probing and manipulating acetogen metabolism, ultimately helping to overcome our dependence on the petrochemical industry.
